# Trends in pancreatic adenocarcinoma incidence and mortality in the United States in the last four decades; a SEER-based study

**DOI:** 10.1186/s12885-018-4610-4

**Published:** 2018-06-25

**Authors:** Anas M. Saad, Tarek Turk, Muneer J. Al-Husseini, Omar Abdel-Rahman

**Affiliations:** 1 Faculty of Medicine, Damascus University, Damascus, Syria; 2 Faculty of Medicine, Damascus University, Damascus, Syria; 3 Faculty of Medicine, Ain Shams University, Cairo, Egypt; 4 Clinical Oncology Department, Faculty of Medicine, Ain Shams University, Lofty Elsayed Street, Cairo, 11566 Egypt; 5 Department of Oncology, University of Calgary and Tom Baker Cancer Center, Calgary, Alberta, Canada

**Keywords:** Incidence, Mortality, Pancreatic adenocarcinoma, SEER

## Abstract

**Background:**

Pancreatic cancer is the fourth-leading cause of cancer deaths in the United States. The silent nature of the disease and its poor prognosis, the need for further research, along with the need to assess the outcomes of current approaches necessitate an ongoing evaluation of the epidemiology and mortality-trends of this malignancy. Continuous monitoring of disease-patterns, on population-levels, may help scientists assess the quality of healthcare delivery, boost their understanding of diseases' characteristics and risk factors, and detect gaps whereby further research is needed. None of the previous reports shed light on pancreatic adenocarcinomas (PAC), the most common type of Pancreatic Cancer, as the primary outcome. In this study we aim to investigate PAC’s incidence and mortality trends over the last four decades in the United States.

**Methods:**

We used SEER 9 database to study PAC cases during 1974-2014. Incidence and mortality rates were calculated by sex, age, race, state and stage of PAC. Annual percent change (APC) was calculated using joinpoint regression software.

**Results:**

We reviewed 67,878 PAC cases; most of these cases were in the head of pancreas. Overall PAC incidence rates increased 1.03% (95% CI, 0.86-1.21, *p* <.001) per year over the study period. Rates of adenocarcinoma of the head of pancreas increased 0.87% (95% CI, 0.68-1.07, *p* <.001), and rates of adenocarcinoma of the body and tail of pancreas increased 3.42% (95% CI, 3.06-3.79, *p* <.001) per year during 1973-2014. PAC incidence-based mortality increased 2.22% (95% CI, 1.93-2.51, *p* <.001) per year. However, during 2012-2014 there was a statistically significant decrease in PAC incidence-based mortality; APC, -24.70% (95% CI, -31.78 - -16.88, *p* <.001).

**Conclusion:**

PAC’s incidence and mortality rates have been increasing for decades. However, the last few years have shown a promising decrease in mortality. We believe that further advances in healthcare delivery and research can lead to a further mortality decrease. Future studies can use this paper as a baseline to keep monitoring the outcomes of PAC's therapy.

**Electronic supplementary material:**

The online version of this article (10.1186/s12885-018-4610-4) contains supplementary material, which is available to authorized users.

## Background

Pancreatic cancer (PC) is an intractable malignancy, and the fourth-leading cause of cancer deaths in the United States, with an estimated of 55 440 new cases, and 44 330 deaths in 2018 [1]. It relatively constitutes a smaller percentage of all cancers' deaths around the globe (7.2%). However, it is one of the most fatal types of cancers with a five-year relative survival rate of 8% [1, 2]. The vast majority (85%) of pancreatic malignancies are adenocarcinomas arising in exocrine glands of the pancreas [3]. Other less common histologies include neuroendocrine tumors such as gastrinoma, insulinoma, somatostatinoma, glucagonoma and non-functional islet cells tumors. Pancreatic adenocarcinoma (PAC) is most commonly diagnosed in the head and neck of the pancreas [2].

At its early stages, pancreatic cancer is usually symptom-free [4]. Upon tumor progression, it manifests as a gradual onset of nonspecific symptoms including jaundice, light-colored stools, abdominal pain, weight loss and fatigue [1]. The available diagnostic tests can also be nonspecific and may miss patients with early stage disease [4]. Surgery, radiotherapy and chemotherapy are traditionally used to extend survival and/or relieve patients' symptoms. However, there is still no definite cure for advanced-stage cases [5]. The silent nature of the disease and its poor prognosis, the need for further research and new local and systemic therapies, along with the need to assess the outcomes of these approaches necessitate an ongoing evaluation of the epidemiology and mortality trends of this malignancy.

The Surveillance, Epidemiology, and End Results (SEER) program of the National Cancer Institute has been collecting data on cancer epidemiology for decades [3]. Such continuous monitoring of disease-patterns, on population-levels, may help scientists to assess the quality of healthcare delivery as well as boost their understanding of diseases' characteristics and risk factors, and detect gaps where further research and interventions are needed [3, 6, 7]. In pancreatic carcinomas, several reports described the trends of incidence and survival [8, 9]. However, these reports varied in conclusions. In addition, recent data show that PC new cases have been rising on average 0.5% each year over the past ten years [3]. Furthermore, none of the previous reports shed light on pancreatic adenocarcinomas as the primary outcome. Therefore, in this study, we aim to add a piece to the puzzle by investigating PAC's incidence and incidence-based mortality trends over the last four decades in the United States.

## Methods

### Data source

We used the SEER*stat software (version 8.3.4) to obtain data of PAC cases diagnosed during 1973-2014 from SEER nine registries [10]. "Incidence - SEER 9 Regs Research Data, Nov 2016 Sub (1973-2014) <Katrina/Rita Population Adjustment>"database covers approximately 9.4% of the US population (based on 2010 census) [11].

### Study population

The study included patients with PAC diagnosed during 1973-2014; ‘Site Recode ICD-O-3/WHO 2008 classification’ and ‘Histology recode - broad groupings’ variables were used for this selection. We excluded cases whose diagnosis relied only on autopsy or death certificates. Within this population, we looked into the following variables: sex, age at diagnosis, race, state, stage at diagnosis (using SEER historic stage A) and site of the tumor within the pancreas (using the ‘primary site’ variable).

### Outcomes

We calculated two main outcomes: incidence and incidence-based mortality rates. All rates were adjusted to the 2000 US standard population and expressed by 100 000 person-years. These rates were calculated during 1973-2014 according to demographic and tumor characteristics. Incidence-based mortality rates were calculated as the number of pancreatic cancer deaths among cases diagnosed over person-time at risk among people in the SEER areas [12]. Rates for Washington and Georgia were calculated starting from 1974 and 1975; respectively, as recording of cases started in these areas after these dates.

Then we calculated the Annual Percentage Changes (APCs) of incidence and incidence-based mortality rates over the study period according to baseline demographic and tumor characteristics.

### Statistical analysis

The SEER*stat software (version 8.3.4) was used to calculate all incidence and incidence-based mortality rates. The National Cancer Institute’s Joinpoint Regression program, version 4.5.0.1 was used to calculate APCs [13]. The Joinpoint Regression software used t tests to determine if APCs were statistically significant from zero; difference was considered statistically significant when *P*< .05. The software analyzed rates over time and detected significant changes in APCs, then selected the best model with the minimum number of joinpoints [14]. All statistical tests were two sided.

## Results

### Baseline characteristics

We reviewed 67 878 PAC cases that were diagnosed from 1973 - 2014 and met our inclusion criteria (Table 1). Most of these cases were white (55 222 patients [81.35%]), older than 60 years (51,573 patients [75.98%]), and had a metastatic cancer (38 852 patients [57.24%]). The most common sub-site for PAC was the head (33 728 patients [49.69%]), followed by the body and tail (14 321 patients [21.1%]). Additional file 1, shows 2014 incidence rates according to demographic and tumor factors. Additional file 2 shows pancreatic adenocarcinoma incidence rates in each individual year from 1973 to 2014.

During the study period, 63 426 eligible cases died of pancreatic cancer and were included in the incidence-based mortality analysis (Table 2). Most of these deaths occurred in whites (51 742 [81.58%]), people older than 60 years (49 994 [78.82%]), and had a metastatic cancer (37 327 [58.85%]). Additional file 3 shows 2014 incidence-based mortality rates according to demographic and tumor factors. Additional file 4 shows pancreatic adenocarcinoma incidence-based mortality rates in each individual year from 1973 to 2014.

### Incidence rates and trends over time

PAC incidence rates were highest among males (8.16 [95% CI, 8.07 - 8.24]), blacks (9.85 [95% CI, 9.63 -10.08]), and people older than 60 years (32.28 [95% CI, 32.00 - 32.56]) (Table 1).

**Table 1:** Pancreatic adenocarcinoma Incidence rates (1973-2014)

Characteristic	Incidence of pancreatic adenocarcinoma		Incidence of adenocarcinoma of the head of pancreas		Incidence of adenocarcinoma of the body and tail of pancreas	
	Cases, No (%)^a^	Rate (95% CI)^b^	Cases, No (%)^a^	Rate (95% CI)^b^	Cases, No (%)^a^	Rate (95% CI)^b^
Overall	67,878 (100)	6.95 (6.90 - 7.00)	33,728 (100)	3.46 (3.42 - 3.50)	14,321 (100)	1.46 (1.43 - 1.48)
Sex						
Male	35,062 (51.65)	8.16 (8.07 - 8.24)	17,033 (50.50)	3.98 (3.92 - 4.04)	7,666 (53.53)	1.76 (1.72 - 1.81)
Female	32,816 (48.35)	5.99 (5.93 - 6.06)	16,695 (49.50)	3.05 (3.00 - 3.09)	6,655 (46.47)	1.22 (1.19 - 1.25)
Race						
White	55,222 (81.35)	6.77 (6.71 - 6.83)	27,492 (81.51)	3.37 (3.33 - 3.42)	11,414 (79.70)	1.40 (1.37 - 1.42)
Black	7,797 (11.49)	9.85 (9.63 -10.08)	3,961 (11.74)	5.02 (4.86 - 5.19)	1,727 (12.06)	2.16 (2.05 - 2.27)
Others^c^	4,755 (7.01)	5.80 (5.63 - 5.97)	2,229 (6.61)	2.72 (2.61 - 2.84)	1,153 (8.05)	1.39 (1.31 - 1.48)
Unknown^d^	104 (0.15)	-	46 (0.14)	-	27 (0.19)	-
Age at diagnosis, y						
<60	16,305 (24.02)	1.93 (1.90 - 1.96)	7,729 (22.92)	0.92 (0.90- 0.94)	3,750 (26.19)	0.44 (0.43 - 0.46)
>60	51,573 (75.98)	32.28 (32.00 - 32.56)	25,999 (77.08)	16.30 (16.10 - 16.50)	10,571 (73.81)	6.58 (6.46 - 6.71)
State^e^						
California	10,960 (16.15)	7.06 (6.93 - 7.20)	5,563 (16.49)	3.60 (3.50 - 3.69)	2,347 (16.39)	1.50 (1.44-1.56)
Connecticut	10,906 (16.07)	7.41 (7.27 - 7.55)	5,363 (15.90)	3.64 (3.54 - 3.74)	2,306 (16.10)	1.57 (1.51-1.63)
Georgia	5,113 (7.53)	7.09 (6.89 - 7.29)	2,585 (7.66)	3.61 (3.46 - 3.75)	1,178 (8.22)	1.61 (1.52-1.71)
Hawaii	2,955 (4.35)	6.47 (6.23 - 6.71)	1,316 (3.90)	2.88 (2.73 - 3.04)	700 (4.89)	1.53 (1.41-1.64)
Iowa	9,079 (13.38)	6.75 (6.61 - 6.89)	4,631 (13.73)	3.42 (3.33 - 3.53)	1,941 (13.55)	1.46 (1.39-1.52)
Michigan	12,539 (18.47)	7.87 (7.73 - 8.01)	6,182 (18.33)	3.89 (3.79 - 3.99)	2,556 (17.85)	1.60 (1.53-1.66)
New Mexico	3,890 (5.73)	6.09 (5.89 - 6.28)	1,928 (5.72)	3.01 (2.87 - 3.15)	671 (4.69)	1.04 (0.96-1.12)
Utah	3,172 (04.67)	5.32 (5.13 - 5.51)	1,581 (4.69)	2.67 (2.54 - 2.81)	667 (4.66)	1.11 (1.02-1.19)
Washington	9,264 (13.65)	6.68 (6.55 - 6.82)	4,579 (13.58)	3.31 (3.22 - 3.41)	1,955 (13.65)	1.40 (1.34-1.47)
Stage at diagnosis^f^						
Localized	5,796 (8.54)	0.60 (0.58 - 0.62)	3,678 (10.90)	0.38 (0.37 - 0.40)	1,123 (7.84)	0.11 (0.11 - 0.12)
Regional	18,623 (27.43)	1.90 (1.88 - 1.93)	13,193 (39.12)	1.35 (1.33 - 1.37)	2,140 (14.94)	0.22 (0.21 - 0.23)
Distant	38,852 (57.24)	3.96 (3.92 - 3.998)	14,685 (43.54)	1.50 (1.47 - 1.52)	10,600 (74.02)	1.08 (1.06 - 1.10)
Unstaged	4,607 (6.79)	0.48 (0.47-0.50)	2,172 (6.44)	0.23 (0.22-0.24)	458 (3.20)	0.05 (0.04-0.05)

^a^Cases included first primary tumors that matched the selection criteria, were microscopically confirmed, and were not identified only from autopsy records

or death certificates

^b^Rates were calculated as number of cases per 100,000 person-years and age adjusted to the 2000 US standard population

^c^Includes American Indian/Alaskan Native and Asian/Pacific Islander

^d^Rates for patients with unknown race could not be calculated, as ‘race’ is a population variable and must be known to calculate rates

^e^Rates were calculated between 1973-2014 for all states except Georgia; 1975-2014, and Washington; 1974-2014

^f^Using SEER historic stage A

PAC incidence rates increased 1.03% (95% CI, 0.86-1.21, *p*<.001) per year over the study period. Rates did not increase significantly during 1983-1999; APC, -0.18% (95% CI,-0.56 - 0.20, *p* = .35), but increased 2.43% (95% CI, 2.11-2.74, *p* <.001) per year during 1999-2014. PAC incidence rates increased for all sex, race, age, state and stage sub-groups. Table 3 describes PAC incidence trends during 1973-2014 by sex, race, age at diagnosis and stage. Additional file 5 summarizes PAC incidence trends by geographical distribution.

**Fig. 1:**
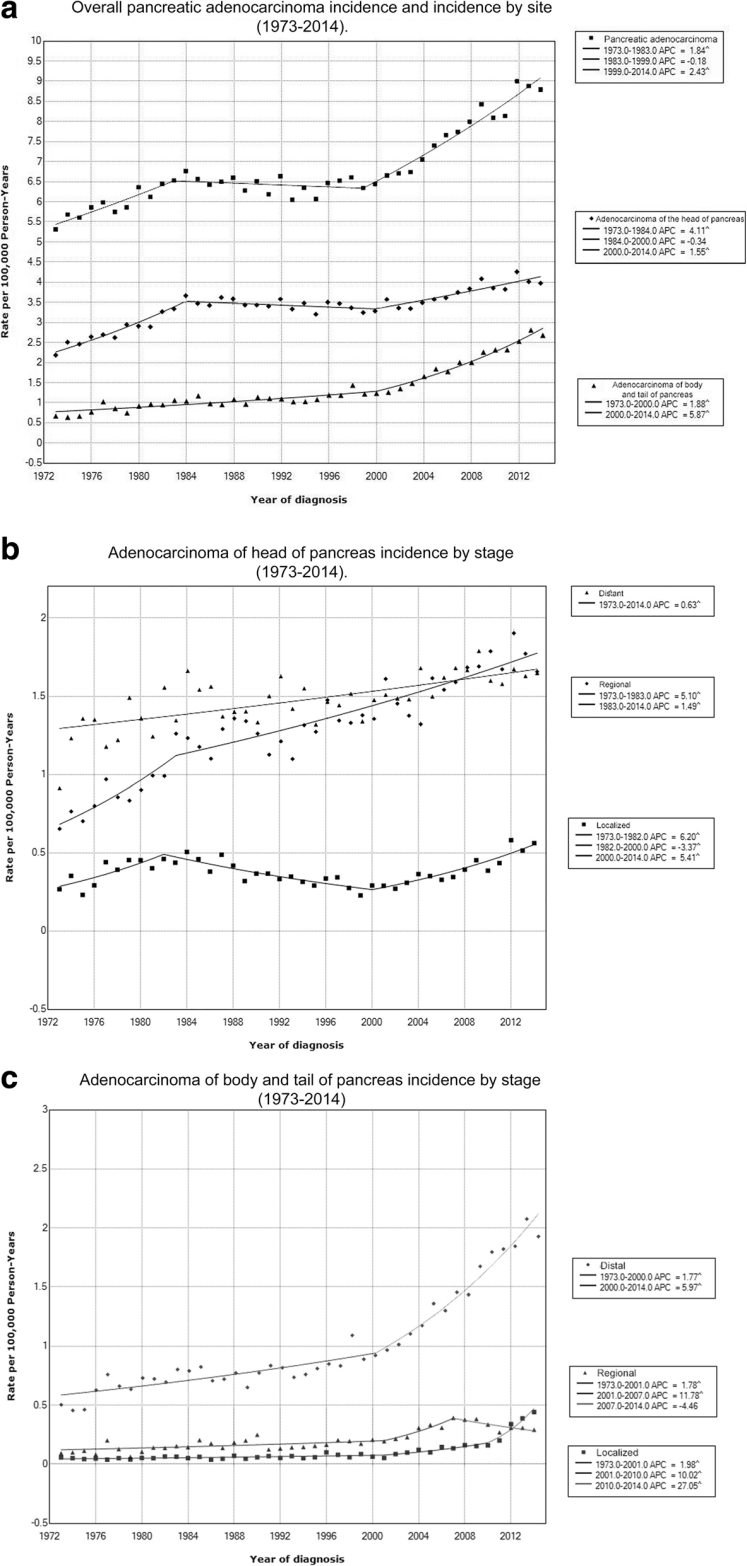
Trends in annual pancreatic adenocarcinoma incidence; **a** according to subsite; **b** according to stage among pancreatic head tumors; **c** according to stage among pancreatic body and tail tumors

Rates of adenocarcinoma of the head of pancreas increased 0.87% (95% CI, 0.68-1.07, *p* <.001), and rates of adenocarcinoma of the body and tail of pancreas increased 3.42% (95% CI, 3.06-3.79, *p* <.001) per year during 1973-2014. Adenocarcinoma of the head of pancreas increased during 1973-2014 for sex, race, age and stage sub-groups except for blacks group and localized stage group, which did not increase significantly. Adenocarcinoma of the body and tail of pancreas increased during 1973-2014 for all sex, race, age and stage sub-groups. Figure 1 shows incidence trends for selected characteristics. Additional file 6 summarizes adenocarcinoma of the head of pancreas, and adenocarcinoma of the body and tail of pancreas incidence trends by sex, race, age at diagnosis and stage.

### Incidence-based mortality rates and trends over times

PAC incidence-based mortality rates were highest among males (7.73 [95% CI, 7.65 - 7.82]), blacks (9.42 [95% CI, 9.19 - 9.64]), and people older than 60 years (31.45 [95% CI, 31.16 - 31.73]) (Table 2).

**Table 2:** Pancreatic adenocarcinoma Incidence-based mortality rates (1973-2014)

characteristic	Incidence-based mortality of pancreatic adenocarcinoma		Incidence-based mortality of adenocarcinoma of the head of pancreas		Incidence-based mortality of adenocarcinoma of the body and tail of pancreas	
	Cases, No (%)^a,b^	Rate (95% CI)^c^	Cases, No (%)^a,b^	Rate (95% CI) ^c^	Cases, No (%)^a,b^	Rate (95% CI) ^c^
Overall	63,426 (100)	6.52 (6.47 - 6.57)	31,609 (100)	3.26 (3.22 - 3.30)	12,859 (100)	1.32 (1.29 - 1.39)
Sex						
Male	32,771 (51.67)	7.73 (7.65 - 7.82)	15,975 (50.54)	3.79 (3.73 - 3.86)	6,903 (53.68)	1.61 (1.57 - 1.65)
Female	30,655 (48.33)	5.58 (5.52 - 5.64)	15,634 (49.46)	2.84 (2.79 - 2.89)	5,956 (46.32)	1.09 (1.06 - 1.11)
Race						
White	51,742 (81.58)	6.36 (6.30 - 6.41)	25,815 (81.67)	3.18 (3.14 - 3.22)	10,289 (80.01)	1.26 (1.24 - 1.28)
Black	7,309 (11.52)	9.42 (9.19 - 9.64)	3,736 (11.82)	4.84 (4.68 – 5.00)	1,557 (12.11)	1.99 (1.89 - 2.09)
Others^d^	4,313 (6.80)	5.34 (5.18 - 5.50)	2,030 (6.42)	2.52 (2.41 - 2.64)	997 (7.75)	1.22 (1.15 - 1.30)
Unknown^e^	62 (0.10)	-	28 (0.09)	-	16 (0.13)	-
Age at death, y						
<60	13,432 (21.18)	1.59 (1.56 - 1.62)	6,358 (20.11)	0.75 (0.73 - 0.77)	2,869 (22.31)	0.34 (0.33 - 0.35)
>60	49,994 (78.82)	31.45 (31.16 - 31.73)	25,251 (79.89)	15.93 (15.73 - 16.13)	9,990 (77.69)	6.25 (6.13 - 6.38)
State^f^						
California	10,192 (16.07)	6.60 (6.47 - 6.73)	5,196 (16.44)	3.38 (3.29 - 3.47)	2,092 (16.27)	1.35 (1.29 - 1.41)
Connecticut	10,146 (16.00)	6.90 (6.76 - 7.03)	4,991 (15.79)	3.39 (3.30 - 3.49)	2,077 (16.15)	1.41 (1.35 - 1.47)
Georgia	4,635 (7.30)	6.57 (6.38 - 6.77)	2,362 (7.47)	3.38 (3.24 - 3.52)	1,012 (7.87)	1.41 (1.33 - 1.51)
Hawaii	2,776 (4.38)	6.11 (5.88 - 6.34)	1,245 (3.94)	2.75 (2.60 - 2.91)	631 (4.91)	1.38 (1.27 - 1.49)
Iowa	8,556 (13.49)	6.31 (6.17 - 6.44)	4,383 (13.87)	3.22 (3.12 - 3.32)	1,767 (13.74)	1.31 (1.25-1.37)
Michigan	11,916 (18.79)	7.52 (7.38 - 7.66)	5,882 (18.61)	3.73 (3.63 - 3.82)	2,350 (18.27)	1.48 (1.42 -1.54)
New Mexico	3,660 (5.77)	5.77 (5.59 - 5.97)	1,817 (5.75)	2.86 (2.73 - 3.00)	613 (4.77)	0.96 (0.88 - 1.04)
Utah	2,948 (4.65)	4.99 (4.81 - 5.17)	1,471 (4.65)	2.51 (2.38 - 2.64)	591 (4.60)	0.99 (0.91 -1.07)
Washington	8,597 (13.55)	6.25 (6.12 - 6.38)	4,262 (13.48)	3.11 (3.02 - 3.21)	1,726 (13.42)	1.25 (1.19-1.31)
Stage at diagnosis^g^						
Localized	4,656 (7.34)	0.49 (0.47 - 0.50)	3,250 (10.28)	0.34 (0.33 - 0.35)	610 (4.74)	0.06 (0.06 - 0.07)
Regional	16,977 (26.77)	1.75 (1.72- 1.78)	12,083 (38.23)	1.25 (1.22 - 1.27)	1,878 (14.60)	0.19 (0.18 - 0.20)
Distant	37,327 (58.85)	3.81 (3.78 - 3.85)	14,170 (44.83)	1.45 (1.42 - 1.47)	9,929 (77.21)	1.01 (0.99 - 1.03)
Unstaged	4,466 (7.04)	0.47 (0.46-0.48)	2,106 (6.66)	0.22 (0.21-0.23)	442 (3.45)	0.05 (0.04-0.05)

^a^Cases included first primary tumors that matched the selection criteria, were microscopically confirmed, and were not identified only from autopsy records or death certificates

^b^No. (%) of deaths were based on cases diagnosed during 1973-2014

^c^Rates were calculated as number of deaths per 100 000 person-years and age adjusted to the 2000 US standard population

^d^Includes American Indian/Alaskan Native and Asian/Pacific Islander

^e^Rates for patients with unknown race could not be calculated, as ‘race’ is a population variable and must be known to calculate rates

^f^Rates were calculated between 1973-2014 for all states except Georgia; 1975-2014, and Washington; 1974-2014

^g^Using SEER historic stage A

**Table 3:** Trends in Pancreatic adenocarcinoma Incidence Rates (1973-2014)

	Overall (1973-2014)		Trends								
			1			2			3		
	APC^a^ (95% CI)	*P* value^b^	year	APC^a^ (95% CI)	*P* value^b^	year	APC^a^ (95% CI)	*P* value^b^	year	APC^a^ (95% CI)	*P* value^b^
Overall	1.03 (0.86-1.21)	<.001	1973-1983	1.84 (0.97-2.71)	<.001	1983-1999	-0.18 (-0.56-0.20)	.35	1999-2014	2.43 (2.11-2.74)	<.001
Sex											
Male	0.88 (0.69-1.06)	<.001	1973-1983	1.10 (0.11-2.09)	.03	1983-1999	-0.25 (-0.69-0.20)	0.27	1999-2014	2.33 (1.97-2.69)	<.001
Female	1.16 (0.97-1.36)	<.001	1973-1984	2.49 (1.40-3.59)	<.001	1984-1999	-0.27 (-0.87-0.34)	0.37	1999-2014	2.54 (2.09-3.00)	<.001
Race											
White	1.09 (0.91-1.28)	<.001	1973-1983	1.77 (0.84-2.70)	<.001	1983-1999	-014 (-0.55-0.28)	0.50	1999-2014	2.59 (2.24-2.95)	<.001
Black	0.65 (0.43-0.87)	<.001	1973-1986	1.85 (0.31-3.40)	.02	1986-1998	-0.73 (-2.20-0.76)	.32	1998-2014	1.47 (0.76-2.18)	<.001
Others^c^	0.89 (0.61-1.18)	<.001	1973-2006	0.26 (-0.09-0.62)	.14	2006-2009	8.41 (-7.86-27.55)	.32	2009-2014	-1.21 (-4.41-2.10)	.46
Age at diagnosis, y											
<60	0.38 (0.18-0.59)	<.001	1973-1984	0.91 (-0.16-1.98)	0.09	1984-1993	-2.14 (-3.76- -0.50)	0.01	1993-2014	1.48 (1.16-1.80)	<.001
>60	1.23 (1.05-1.40)	<.001	1973-1984	2.11 (1.32-2.90)	<.001	1984-1999	-0.07 (-0.50-0.36)	0.74	1999-2014	2.58 (2.26-2.91)	<.001
Stage at diagnosis^d^											
Localized	1.67 (1.03-2.32)	<.001	1973-1981	6.05 (1.61-10.69)	.01	1981-2001	-2.11 (-3.06 - -1.16)	<.001	2001-2014	7.98 (6.63-9.35)	<.001
Regional	1.84 (1.63-2.04)	<.001	1973-2014	1.84 (1.63-2.04)	<.001						
Distant	0.95 (0.73-1.18)	<.001	1973-1976	7.56 (-0.44-16.41)	0.06	1976-1995	-0.55 (-0.96- -0.14)	0.01	1995-2014	2.20 (1.90-2.50)	<.001

^a^Annual Percentage Changes, calculated using Joinpoint regression software

^b^Two-sided P value was calculated using t test to determine the significance of APC change

^c^Includes American Indian/Alaskan Native and Asian/Pacific Islander

^d^Using SEER historic stage A

PAC incidence-based mortality increased 2.22% (95% CI, 1.93-2.51, *p* <.001) per year over the study period. However, during 2012-2014 there was a statistically significant decrease in PAC incidence-based mortality; APC, -24.70% (95% CI, -31.78 - -16.88, *p* <.001). PAC incidence-based mortality rates increased for all sex, race, age, state and stage sub-groups during the study period. Interestingly, incidence based-mortality rates decreased between 2012 and 2014 in all states except Georgia, Hawaii and Utah. Table 4 describes PAC incidence-based mortality trends during 1973-2014 by sex, race, age at death and stage. Additional file 7 summarizes PAC incidence-based mortality trends by geographical distribution.

**Table 4:** Trends in Pancreatic Cancer Incidence-based mortality Rates (1973-2014)

	Overall (1973-2014)		Trend											
			1			2			3			4		
	APC^a^ (95% CI)	*P* value^b^	Year	APC^a^ (95% CI)	*P* value^b^	Year	APC^a^ (95% CI)	*P* value^b^	Year	APC^a^ (95% CI)	*P* value^b^	Year	APC^a^ (95% CI)	*P* value^b^
Overall	2.22 (1.93-2.51)	<.001	1973-1982	4.58 (3.38-5.79)	<.001	1982-2000	1.45 (1.08-1.83)	<.001	2000-2012	3.83 (3.21-4.45)	<.001	2012-2014	-24.70 (-31.78 - -16.88)	<.001
Sex														
Male	2.31 (2.03-2.59)	<.001	1973-1975	15.14 (0.57-31.82)	.04	1975-2000	1.83 (1.59-2.06)	<.001	2000-2012	4.03 (3.40-4.67)	<.001	2012-2014	-24.52 (-31.71 - -16.57)	<.001
Female	2.17 (1.85-2.49)	<.001	1973-1983	5.34 (3.99-6.71)	<.001	1983-2000	1.20 (0.68-1.73)	<.001	2000-2012	3.66 (2.85-4.47)	<.001	2012-2014	-24.79 (-33.89 - -14.44)	<.001
Race														
White	1.92 (1.64-2.21)	<.001	1973-1976	12.10 (4.45-20.30)	<.001	1976-2001	1.53 (1.29-1.78)	<.001	2001-2012	3.50 (2.73-4.28)	<.001	2012-2014	-24.89 (-32.58 - -16.32)	<.001
Black	2.91 (2.54-3.27)	<.001	1973-1980	8.93 (4.65-13.38)	<.001	1980-2001	2.19 (1.55-2.82)	<.001	2001-2012	4.46 (3.00-5.94)	<.001	2012-2014	-24.30 (-37.98 - -7.60)	.01
Others^c^	4.86 (4.40-5.31)	<.001	1973-2012	5.23 (4.92-5.53)	<.001	2012-2014	-22.08 (-37.33 - -3.13)	.03						
Age at death, y														
<60	1.50 (1.10-1.91)	<.001	1973-1993	0.19 (-0.34-0.73)	.46	1993-2005	4.03 (2.84-5.24)	<.001	2005-2012	0.44 (-2.06-3.00)	.72	2012-2014	-33.46 (-45.65 - -18.53)	<.001
>60	2.40 (2.10-2.70)	<.001	1973-1983	5.42 (4.30-6.55)	<.001	1983-2002	1.46 (1.11-1.82)	<.001	2002-2012	4.57 (3.69-5.46)	<.001	2012-2014	-22.92 (-30.31 - -14.75)	<.001
Stage at diagnosis^d^														
Localized	1.39 (0.92-1.87)	<.001	1973-1981	9.30 (5.75-12.97)	<.001	1981-1999	-0.94 (-1.83 - -0.04)	.04	1999-2012	4.57 (3.09-6.07)	<.001	2012-2014	-27.92 (-44.80 - -5.87)	.02
Regional	2.94 (2.47-3.41)	<.001	1973-1977	12.80 (2.50-24.14)	.02	1977-2012	3.13 (2.88-3.38)	<.001	2012-2014	-36.81 (-49.61 - -20.77)	<.001			
Distant	2.35 (2.07-2.63)	<.001	1973-1975	25.14 (6.66-46.83)	.01	1975-1995	1.10 (0.72-1.49)	<.001	1995-2012	3.83 (3.42-4.25)	<.001	2012-2014	-17.61 (-25.68 - -8.66)	<.001

^a^Annual Percentage Changes, calculated using Joinpoint regression software

^b^Two-sided P value was calculated using t test to determine the significance of APC change

^c^Includes American Indian/Alaskan Native and Asian/Pacific Islander

^d^Using SEER historic stage A

**Fig. 2:**
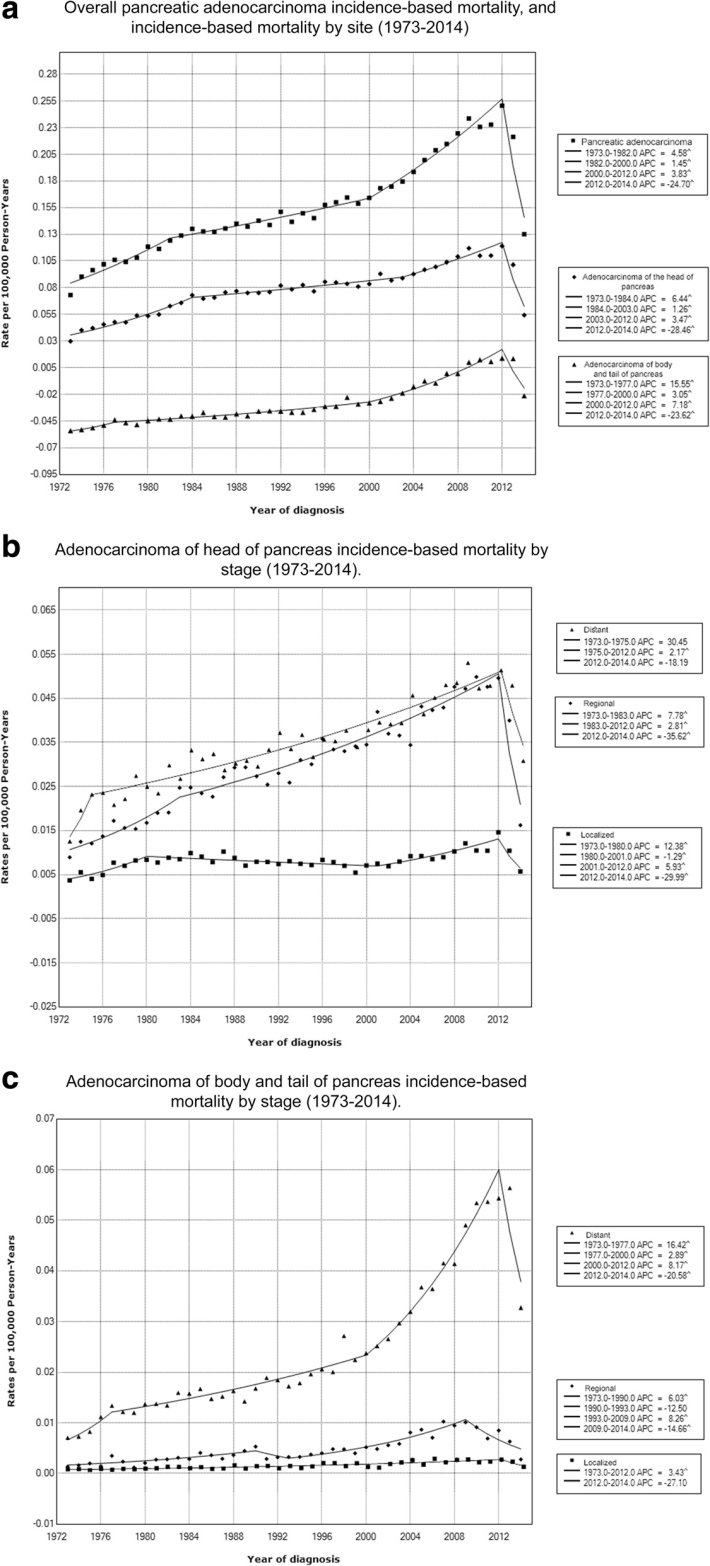
Trends in annual pancreatic adenocarcinoma incidence-based mortality; **a** according to subsite; **b** according to stage among pancreatic head tumors; **c** according to stage amog pancreatic body and tail tumors

During 1973-2014 there was a statistically significant increase in mortality rates of adenocarcinoma of the head of pancreas; APC, 2.11% (95% CI, 1.73-2.50, *p* <.001), and adenocarcinoma of body and tail of pancreas; APC, 4.31% (95% CI, 3.88-4.74, *p* <.001). Incidence-based mortality rates of both adenocarcinoma of head of pancreas, and adenocarcinoma of body and tail increased in all sex, race, age, and stage sub-groups during 1973-2014. Figure 2 shows incidence-based mortality trends for selected characteristics. Additional file 6 summarizes adenocarcinoma of the head of pancreas, and adenocarcinoma of the body and tail of pancreas incidence trends by sex, race, age at diagnosis and stage.

## Discussion

To our knowledge, this is the first study to outline the trends of PAC during the past four decades. Our results revealed an overall increase in incidence and incidence-based mortality rates of PAC during the study period.

Being a rapidly fatal malignancy, PAC represents a challenge for clinicians in terms of early detection and management [15]. Such a poor prognosis can be attributed to the relatively silent progression of pancreatic tumors at early stages; the tumor usually invades locally and/or spreads before diagnosis [15]. Consequently, this tumor's mortality rates are notably close to its incidence rates [9]. Few studies have investigated the temporal trends of pancreatic cancer with its incidence and mortality [8, 9, 16–18]. Besides few variations, results from these studies have almost always showed an increase in all types of pancreatic cancer incidence and mortality, which is consistent with our results on PAC. This continuous increase draws attention to the need for more research and efforts on preventive measures to battle PCs. Besides smoking cessation, several lifestyle changes were recommended as preventive measures including controlled alcohol consumption, weight loss and consuming more fruit and whole grains [19]. However, there is still no level I evidence supporting the efficacy of these recommendations.

Calculating incidence based-mortality (IBM) using population-based registries allows partitioning of mortality by variables associated with the cancer onset [12, 13]. In addition, it can reflect the effectiveness of present treatment modalities. For PAC, surgery is the mainstay treatment in resectable tumors (+/- perioperative treatment). A recent review demonstrated significant advances in pancreatic cancer treatment on different levels including surgical technology, imaging technology and systemic therapy regimens [5]. These advances could explain the significant decrease in incidence based-mortality (IBM) rates between 2012 and 2014 that was reported in our study. These results, along with the promising research on targeted therapy, highlight the importance of continuous monitoring and updating of PAC IBM rates to assess the implication of clinical approaches. This result could also be, potentially, attributed to the recent introduction of the FOLFIRINOX regimen, which is a combination of several chemotherapy agents (Fluorouracil 5-FU; Leucovorin; Irinotecan; Oxaliplatin) that was presented at the 2010 American Society of Clinical Oncology (ASCO) meeting [20]. However, there is still not hard evidence to support this claim, and further research is required in this context.

Despite the recent advances in molecular understanding of PAC, scientists still lack a full picture on its etiology. However, several risk factors were linked to a higher risk of pancreatic neoplasia [21]. Tobacco smoking represents the most well-established risk factor, with an estimated two-fold risk of pancreatic malignancy in smokers compared to nonsmokers [22, 23]. However, researchers argue that smoking alone does not sufficiently explain the variation in pancreatic cancer's incidence around the globe, and that more attention should be paid to other risk factors such as diet and hormonal influences in addition to certain strain types of H. Pylori [9, 21, 24, 25]. Generally, a more in-depth understanding of the trends of PAC can play role in assessing and investigating these risk agents, for instance, although Risch et al found that H.Pylori CagA Strain type might increase the incidence of PC, other researchers like Schulte et al in their meta-analysis concluded that there was no overall relationship between H. Pylori and pancreatic cancer [25, 26]. Unfortunately, as reported in previous SEER-based studies on pancreatic cancer, we could not evaluate the effect of these factors due to the lack of such data in the available registries [9].

Although pancreatic masses in any site must trigger a red alarm, the primary location of a tumor can be a valuable indicator of a tumor's prognosis [27]. It is also pivotal to plan any surgical and non-surgical approach. Previous reports on pancreatic malignancies have shown the head of pancreas to be the most common site; our results in PAC are consistent with these results [8, 9]. However, the increase in body and tail tumors over the past four decades is statistically higher than in-head tumors, which was also found by Lau *et al* in their study over the period from 1973 till 2002 [8]. The latest finding could be one of the interpretations to the increased IBM since body and tail tumors are usually associated with poor prognosis due to their late presentation [28].

Like risk factors and tumor locations, geographic distribution of incidence and mortality rates adds valuable information to the epidemiological characteristics of diseases. Our results showed that Georgia, Hawaii and Utah were the only states where incidence-based mortality rates did not decrease in 2012-2014. The reason behind this finding is yet to be investigated. It can be due to fewer cases in these states that hindered the detection of a significant decrease in incidence based-mortality (IBM), to different quality of healthcare delivery, or can even be related to the characteristics of patients in these states and their health-awareness and lifestyle.

This study, like other SEER-based studies, is limited by the availability of data in the registries. For instance, some analyses and comparisons may not be feasible due to the unavailability or incompleteness of certain variables. In addition, the course of treatment and the factors that lead to a certain approach may also be missing. All details related to SEER-related limitations are demonstrated in separate reports [29, 30].

## Conclusion

In summary, PAC's incidence and mortality rates have been increasing for decades, and it is expected to become the second leading cause of cancer-related death in the US by 2030 [31]. However, the past few years have shown a promising decrease in mortality. Further advances in healthcare delivery and research can lead to a further mortality decrease. Healthcare professionals and policy-makers can also make more efforts to control the associated risk factors, especially smoking. These efforts could range from awareness campaigns and advocating for lifestyle changes to imposing more strict smoking-related laws. All these attempts, along with persistent monitoring of the outcomes, can help to tackle the burden of PAC on a global scale.

## Additional files

Additional file 1: Pancreatic adenocarcinoma Incidence rates (2014). (DOCX 13 kb)

Additional file 2: Pancreatic adenocarcinoma Incidence rates for each individual year (1973-2014). (DOCX 14 kb)

Additional file 3: Pancreatic adenocarcinoma Incidence-based mortality rates (2014). (DOCX 13 kb)

Additional file 4: Pancreatic adenocarcinoma Incidence-based mortality rates for each individual year (1973-2014). (DOCX 14 kb)

Additional file 5: Trends in Pancreatic adenocarcinoma Incidence Rates by state (1973-2014) (DOCX 13 kb)

Additional file 6: Trends in Adenocarcinoma of head of pancreas and Adenocarcinoma of body and tail of pancreas Incidence and incidence-based mortality rates (1973-2014). (DOCX 13 kb)

Additional file 7: Trends in Pancreatic adenocarcinoma Incidence-based mortality Rates by state (1973-2014). (DOCX 13 kb)

## Abbreviations

APC: Annual percent change
IBM: Incidence based-mortality
PAC: Pancreatic adenocarcinomas
PC: Pancreatic cancer
SEER: The Surveillance, Epidemiology, and End Results
