# Supplementary material for: Trends in pancreatic adenocarcinoma incidence and mortality in the United States in the last four decades; a SEER-based study
Source: BMC Cancer. 2018 Jun 25;18:688. doi: 10.1186/s12885-018-4610-4 (PMC6020186; doi:10.1186/s12885-018-4610-4)
Supplement: Supplementary file 1 — Pancreatic adenocarcinoma Incidence rates (2014). (DOCX 13 kb) [file 12885_2018_4610_MOESM1_ESM.docx]

Additional file 1. Pancreatic adenocarcinoma Incidence rates (2014)

| characteristic | 2014 Incidence of pancreatic adenocarcinoma | | 2014 Incidence of adenocarcinoma of the head of pancreas | | 2014 Incidence of adenocarcinoma of the body and tail of pancreas | |
| --- | --- | --- | --- | --- | --- | --- |
|  | Cases, No^a^ | Rate^b^ | Cases, No^a^ | Rate^b^ | Cases, No^a^ | Rate^b^ |
| Overall | 2,940 | 8.79 | 1,342 | 3.98 | 898 | 2.68 |
| Sex |  |  |  |  |  |  |
| Male | 1,565 | 10.32 | 693 | 4.55 | 495 | 3.24 |
| Female | 1,375 | 7.55 | 649 | 3.52 | 403 | 2.23 |
| Race |  |  |  |  |  |  |
| White | 2,304 | 8.81 | 1,057 | 4.01 | 700 | 2.67 |
| Black | 363 | 11.28 | 168 | 5.10 | 115 | 3.50 |
| Others^c^ | 255 | 6.31 | 110 | 2.67 | 78 | 1.97 |
| Age at diagnosis, y |  |  |  |  |  |  |
| <60 | 1,056 | 3.20 | 493 | 1.48 | 345 | 1.06 |
| >60 | 1,884 | 47.46 | 849 | 21.27 | 553 | 13.85 |
| State^d^ |  |  |  |  |  |  |
| California | 433 | 8.29 | 198 | 3.64 | 146 | 2.83 |
| Connecticut | 445 | 9.98 | 206 | 4.52 | 118 | 2.74 |
| Georgia | 325 | 10.54 | 139 | 4.54 | 111 | 3.52 |
| Hawaii | 113 | 6.54 | 38 | 2.21 | 36 | 2.18 |
| Iowa | 372 | 9.81 | 174 | 4.58 | 112 | 2.94 |
| Michigan | 449 | 9.72 | 206 | 4.39 | 136 | 2.92 |
| New Mexico | 166 | 6.58 | 76 | 2.99 | 38 | 1.50 |
| Utah | 171 | 6.86 | 82 | 3.40 | 54 | 2.12 |
| Washington | 466 | 8.60 | 223 | 4.05 | 147 | 2.70 |
| Stage at diagnosis^e^ |  |  |  |  |  |  |
| Localized | 390 | 1.18 | 188 | 0.56 | 145 | 0.44 |
| Regional | 809 | 2.42 | 557 | 1.66 | 97 | 0.29 |
| Distant | 1,671 | 4.98 | 560 | 1.65 | 650 | 1.93 |

^a^ Cases included first primary tumors that matched the selection criteria, were microscopically confirmed, and were not identified only from autopsy records or death certificates.

^b^ Rates were calculated as number of cases per 100,000 person-years and age adjusted to the 2000 US standard population.

^c^ Includes American Indian/Alaskan Native and Asian/Pacific Islander.

^d^ rates were calculated between 1973-2014 for all states except Georgia; 1975-2014, and Washington; 1974-2014

^e^ using SEER historic stage A
