# Supplementary material for: Trends in pancreatic adenocarcinoma incidence and mortality in the United States in the last four decades; a SEER-based study
Source: BMC Cancer. 2018 Jun 25;18:688. doi: 10.1186/s12885-018-4610-4 (PMC6020186; doi:10.1186/s12885-018-4610-4)
Supplement: Supplementary file 2 — Pancreatic adenocarcinoma Incidence rates for each individual year (1973-2014). (DOCX 14 kb) [file 12885_2018_4610_MOESM2_ESM.docx]

Additional file 2. Pancreatic adenocarcinoma Incidence rates for each individual year (1973-2014)

| characteristic | Incidence of pancreatic adenocarcinoma | | Incidence of adenocarcinoma of the head of pancreas | | Incidence of adenocarcinoma of the body and tail of pancreas | |
| --- | --- | --- | --- | --- | --- | --- |
|  | Cases, No^a^ | Rate (95% CI)^b^ | Cases, No^a^ | Rate (95% CI)^b^ | Cases, No^a^ | Rate (95% CI)^b^ |
| 1973 | 732 | 5.31 | 297 | 2.19 | 94 | 0.67 |
| 1974 | 898 | 5.68 | 398 | 2.51 | 103 | 0.64 |
| 1975 | 960 | 5.61 | 415 | 2.46 | 117 | 0.66 |
| 1976 | 1,015 | 5.86 | 451 | 2.64 | 138 | 0.77 |
| 1977 | 1,053 | 5.98 | 477 | 2.70 | 177 | 1.02 |
| 1978 | 1,040 | 5.75 | 475 | 2.62 | 158 | 0.86 |
| 1979 | 1,068 | 5.86 | 531 | 2.95 | 142 | 0.75 |
| 1980 | 1,175 | 6.36 | 529 | 2.91 | 172 | 0.92 |
| 1981 | 1,149 | 6.12 | 544 | 2.89 | 184 | 0.97 |
| 1982 | 1,224 | 6.45 | 618 | 3.27 | 182 | 0.95 |
| 1983 | 1,266 | 6.53 | 645 | 3.34 | 210 | 1.06 |
| 1984 | 1,320 | 6.76 | 710 | 3.67 | 204 | 1.04 |
| 1985 | 1,303 | 6.57 | 688 | 3.47 | 232 | 1.17 |
| 1986 | 1,301 | 6.42 | 698 | 3.43 | 200 | 0.98 |
| 1987 | 1,333 | 6.50 | 741 | 3.62 | 198 | 0.95 |
| 1988 | 1,378 | 6.60 | 750 | 3.59 | 229 | 1.08 |
| 1989 | 1,337 | 6.28 | 732 | 3.44 | 207 | 0.97 |
| 1990 | 1,395 | 6.51 | 734 | 3.44 | 245 | 1.14 |
| 1991 | 1,353 | 6.19 | 741 | 3.41 | 241 | 1.11 |
| 1992 | 1,480 | 6.64 | 802 | 3.58 | 245 | 1.10 |
| 1993 | 1,372 | 6.05 | 755 | 3.33 | 235 | 1.02 |
| 1994 | 1,455 | 6.35 | 801 | 3.48 | 235 | 1.03 |
| 1995 | 1,415 | 6.07 | 747 | 3.20 | 253 | 1.08 |
| 1996 | 1,529 | 6.47 | 828 | 3.51 | 281 | 1.19 |
| 1997 | 1,569 | 6.53 | 834 | 3.47 | 284 | 1.18 |
| 1998 | 1,613 | 6.61 | 820 | 3.36 | 351 | 1.44 |
| 1999 | 1,571 | 6.34 | 803 | 3.25 | 302 | 1.22 |
| 2000 | 1,618 | 6.44 | 824 | 3.28 | 310 | 1.23 |
| 2001 | 1,694 | 6.65 | 910 | 3.57 | 321 | 1.26 |
| 2002 | 1,736 | 6.71 | 869 | 3.36 | 352 | 1.35 |
| 2003 | 1,774 | 6.74 | 881 | 3.35 | 390 | 1.48 |
| 2004 | 1,886 | 7.05 | 933 | 3.50 | 443 | 1.65 |
| 2005 | 2,023 | 7.40 | 975 | 3.57 | 504 | 1.84 |
| 2006 | 2,117 | 7.66 | 1,003 | 3.62 | 489 | 1.78 |
| 2007 | 2,193 | 7.74 | 1,061 | 3.75 | 569 | 2.01 |
| 2008 | 2,193 | 7.99 | 1,115 | 3.84 | 586 | 2.00 |
| 2009 | 2,491 | 8.43 | 1,212 | 4.09 | 667 | 2.26 |
| 2010 | 2,465 | 8.09 | 1,169 | 3.86 | 714 | 2.32 |
| 2011 | 2,522 | 8.14 | 1,183 | 3.83 | 720 | 2.32 |
| 2012 | 2,889 | 9.00 | 1,364 | 4.26 | 822 | 2.53 |
| 2013 | 2,900 | 8.88 | 1,323 | 4.02 | 917 | 2.81 |
| 2014 | 2,940 | 8.79 | 1,342 | 3.98 | 898 | 2.68 |

^a^ Cases included first primary tumors that matched the selection criteria, were microscopically confirmed, and were not identified only from autopsy records or death certificates.

^b^ Rates were calculated as number of cases per 100,000 person-years and age adjusted to the 2000 US standard population.
