# Supplementary material for: Trends in pancreatic adenocarcinoma incidence and mortality in the United States in the last four decades; a SEER-based study
Source: BMC Cancer. 2018 Jun 25;18:688. doi: 10.1186/s12885-018-4610-4 (PMC6020186; doi:10.1186/s12885-018-4610-4)
Supplement: Supplementary file 3 — Pancreatic adenocarcinoma Incidence-based mortality rates (2014). (DOCX 13 kb) [file 12885_2018_4610_MOESM3_ESM.docx]

Additional file 3. Pancreatic adenocarcinoma Incidence-based mortality rates (2014)

| characteristic | 2014 Incidence-based mortality of pancreatic adenocarcinoma | | 2014 Incidence-based mortality of adenocarcinoma of the head of pancreas | | 2014 Incidence-based mortality of adenocarcinoma of the body and tail of pancreas | |
| --- | --- | --- | --- | --- | --- | --- |
|  | Cases, No^a,b^ | Rate^c^ | Cases, No^a,b^ | Rate^c^ | Cases, No^a,b^ | Rate^c^ |
| Overall | 1,252 | 0.13 | 523 | 0.05 | 361 | 0.04 |
| Sex |  |  |  |  |  |  |
| Male | 658 | 0.16 | 249 | 0.06 | 211 | 0.05 |
| Female | 594 | 0.11 | 274 | 0.05 | 150 | 0.03 |
| Race |  |  |  |  |  |  |
| White | 977 | 0.12 | 403 | 0.05 | 282 | 0.03 |
| Black | 164 | 0.22 | 73 | 0.10 | 52 | 0.07 |
| Others^d^ | 108 | 0.14 | 46 | 0.06 | 26 | 0.03 |
| Age at death, y |  |  |  |  |  |  |
| <60 | 347 | 0.04 | 150 | 0.02 | 105 | 0.01 |
| >60 | 905 | 0.77 | 373 | 0.32 | 256 | 0.22 |
| State^e^ |  |  |  |  |  |  |
| California | 171 | 0.11 | 74 | 0.05 | 57 | 0.04 |
| Connecticut | 165 | 0.11 | 65 | 0.04 | 38 | 0.03 |
| Georgia | 143 | 0.21 | 53 | 0.08 | 54 | 0.08 |
| Hawaii | 50 | 0.11 | 16 | 0.04 | 10 | 0.02 |
| Iowa | 157 | 0.11 | 66 | 0.05 | 50 | 0.04 |
| Michigan | 221 | 0.14 | 94 | 0.06 | 60 | 0.04 |
| New Mexico | 73 | 0.12 | 30 | 0.05 | 15 | 0.02 |
| Utah | 79 | 0.13 | 35 | 0.06 | 26 | 0.04 |
| Washington | 193 | 0.14 | 90 | 0.07 | 51 | 0.04 |
| Stage at diagnosis^f^ |  |  |  |  |  |  |
| Localized | 78 | 0.01 | 53 | 0.01 | 12 | 0.00 |
| Regional | 219 | 0.02 | 154 | 0.02 | 26 | 0.00 |
| Distant | 925 | 0.10 | 301 | 0.03 | 321 | 0.03 |

^a^ Cases included first primary tumors that matched the selection criteria, were microscopically confirmed, and were not identified only from autopsy records or death certificates.

^b^ No. (%) of deaths were based on cases diagnosed during 1973-2014

^c^ Rates were calculated as number of deaths per 100 000 person-years and age adjusted to the 2000 US standard population.

^d^ Includes American Indian/Alaskan Native and Asian/Pacific Islander.

^e^ rates were calculated between 1973-2014 for all states except Georgia; 1975-2014, and Washington; 1974-2014

^f^ using SEER historic stage A
