# Supplementary material for: Trends in pancreatic adenocarcinoma incidence and mortality in the United States in the last four decades; a SEER-based study
Source: BMC Cancer. 2018 Jun 25;18:688. doi: 10.1186/s12885-018-4610-4 (PMC6020186; doi:10.1186/s12885-018-4610-4)
Supplement: Supplementary file 4 — Pancreatic adenocarcinoma Incidence-based mortality rates for each individual year (1973-2014). (DOCX 14 kb) [file 12885_2018_4610_MOESM4_ESM.docx]

Additional file 4. Pancreatic adenocarcinoma Incidence-based mortality rates for each individual year (1973-2014)

| year | Incidence-based mortality of pancreatic adenocarcinoma | | Incidence-based mortality of adenocarcinoma of the head of pancreas | | Incidence-based mortality of adenocarcinoma of the body and tail of pancreas | |
| --- | --- | --- | --- | --- | --- | --- |
|  | Cases, No^a,b^ | Rate^c^ | Cases, No^a,b^ | Rate^c^ | Cases, No^a,b^ | Rate^c^ |
| 1973 | 730 | 0.07 | 296 | 0.03 | 94 | 0.01 |
| 1974 | 898 | 0.09 | 398 | 0.04 | 103 | 0.01 |
| 1975 | 956 | 0.10 | 415 | 0.04 | 116 | 0.01 |
| 1976 | 1,012 | 0.10 | 450 | 0.05 | 138 | 0.01 |
| 1977 | 1,051 | 0.11 | 476 | 0.05 | 176 | 0.02 |
| 1978 | 1,038 | 0.10 | 473 | 0.05 | 158 | 0.02 |
| 1979 | 1,066 | 0.11 | 529 | 0.05 | 142 | 0.01 |
| 1980 | 1,171 | 0.12 | 528 | 0.05 | 171 | 0.02 |
| 1981 | 1,146 | 0.12 | 541 | 0.05 | 184 | 0.02 |
| 1982 | 1,220 | 0.12 | 615 | 0.06 | 181 | 0.02 |
| 1983 | 1,265 | 0.13 | 645 | 0.07 | 209 | 0.02 |
| 1984 | 1,320 | 0.14 | 710 | 0.07 | 204 | 0.02 |
| 1985 | 1,299 | 0.13 | 684 | 0.07 | 232 | 0.02 |
| 1986 | 1,295 | 0.13 | 696 | 0.07 | 200 | 0.02 |
| 1987 | 1,321 | 0.14 | 733 | 0.08 | 198 | 0.02 |
| 1988 | 1,367 | 0.14 | 749 | 0.08 | 224 | 0.02 |
| 1989 | 1,334 | 0.14 | 731 | 0.08 | 205 | 0.02 |
| 1990 | 1,385 | 0.14 | 727 | 0.08 | 244 | 0.02 |
| 1991 | 1,340 | 0.14 | 735 | 0.08 | 238 | 0.02 |
| 1992 | 1,467 | 0.15 | 795 | 0.08 | 242 | 0.02 |
| 1993 | 1,361 | 0.14 | 751 | 0.08 | 232 | 0.02 |
| 1994 | 1,439 | 0.15 | 793 | 0.08 | 229 | 0.02 |
| 1995 | 1,404 | 0.15 | 740 | 0.08 | 252 | 0.03 |
| 1996 | 1,518 | 0.16 | 822 | 0.09 | 277 | 0.03 |
| 1997 | 1,537 | 0.16 | 815 | 0.08 | 276 | 0.03 |
| 1998 | 1,589 | 0.16 | 808 | 0.08 | 347 | 0.04 |
| 1999 | 1,535 | 0.16 | 784 | 0.08 | 293 | 0.03 |
| 2000 | 1,583 | 0.16 | 806 | 0.08 | 302 | 0.03 |
| 2001 | 1,657 | 0.17 | 888 | 0.09 | 312 | 0.03 |
| 2002 | 1,688 | 0.17 | 840 | 0.09 | 340 | 0.03 |
| 2003 | 1,731 | 0.18 | 859 | 0.09 | 377 | 0.04 |
| 2004 | 1,823 | 0.19 | 897 | 0.09 | 428 | 0.04 |
| 2005 | 1,936 | 0.20 | 938 | 0.10 | 473 | 0.05 |
| 2006 | 2,010 | 0.21 | 958 | 0.10 | 452 | 0.05 |
| 2007 | 2,085 | 0.21 | 1,005 | 0.10 | 533 | 0.05 |
| 2008 | 2,188 | 0.22 | 1,058 | 0.11 | 534 | 0.05 |
| 2009 | 2,312 | 0.24 | 1,132 | 0.12 | 614 | 0.06 |
| 2010 | 2,247 | 0.23 | 1,063 | 0.11 | 641 | 0.07 |
| 2011 | 2,264 | 0.23 | 1,066 | 0.11 | 624 | 0.06 |
| 2012 | 2,439 | 0.25 | 1,152 | 0.12 | 657 | 0.07 |
| 2013 | 2,147 | 0.22 | 985 | 0.10 | 646 | 0.07 |
| 2014 | 1,252 | 0.13 | 523 | 0.05 | 361 | 0.04 |

^a^ Cases included first primary tumors that matched the selection criteria, were microscopically confirmed, and were not identified only from autopsy records or death certificates.

^b^ No of deaths were based on cases diagnosed during 1973-2014

^c^ Rates were calculated as number of deaths per 100 000 person-years and age adjusted to the 2000 US standard population
