# Supplementary material for: Trends in pancreatic adenocarcinoma incidence and mortality in the United States in the last four decades; a SEER-based study
Source: BMC Cancer. 2018 Jun 25;18:688. doi: 10.1186/s12885-018-4610-4 (PMC6020186; doi:10.1186/s12885-018-4610-4)
Supplement: Supplementary file 5 — Trends in Pancreatic adenocarcinoma Incidence Rates by state (1973-2014) (DOCX 13 kb) [file 12885_2018_4610_MOESM5_ESM.docx]

Additional file 5. Trends in Pancreatic adenocarcinoma Incidence Rates by state (1973-2014)

|  | Overall  (1973-2014)^a^ | | Trends | | | | | | | | |  |
| --- | --- | --- | --- | --- | --- | --- | --- | --- | --- | --- | --- | --- |
|  |  |  | 1 | | | 2 | | | 3 | | | |
|  | APC^b^  (95% CI) | P value^c^ | year | APC^b^  (95% CI) | P value^c^ | year | APC^b^  (95% CI) | P value^c^ | year | APC^b^  (95% CI) | P value^c^ | |
| California | 0.31  (0.06-0.55) | .02 | 1973-1975 | 10.91  (-10.79-37.91) | .34 | 1975-2001 | -0.63  (-1.01- -0.25) | <.001 | 2001-2014 | 2.36  (1.52-3.21) | <.001 | |
| Connecticut | 1.45  (1.21-1.68) | <.001 | 1973-1992 | 0.46  (-0.34-1.26) | .25 | 1992-2014 | 2.07  (1.58-2.57) | <.001 |  |  |  | |
| Georgia | 1.65  (1.29-2.02) | <.001 | 1975-1996 | 0.39  (-0.65-1.45) | .45 | 1996-2014 | 2.70  (1.83-3.57) | <.001 |  |  |  | |
| Hawaii | 0.47  (0.13-0.82) | .01 | 1973-2006 | -0.12  (-0.55-0.31) | .57 | 2006-2012 | 5.54  (0.24-11.12) | .04 | 2012-2014 | -11.79  (-29.13-9.79) | .25 | |
| Iowa | 1.50  (1.24-1.75) | <.001 | 1973-1984 | 2.69  (1.10-4.30) | <.001 | 1984-1995 | -0.79  (-2.47-0.91) | .35 | 1995-2014 | 2.73  (2.17-3.29) | <.001 | |
| Michigan | 1.28  (1.06-1.50) | <.001 | 1973-1985 | 4.17  (3.10-5.26) | <.001 | 1985-1993 | -1.72  (-3.70-0.30) | .09 | 1993-2014 | 1.80  (1.45-2.14) | <.001 | |
| New Mexico | 0.63  (0.19-1.06) | .01 | 1973-2003 | -0.17  (-0.89-0.55) | .63 | 2003-2014 | 3.05  (0.80-5.35) | .01 |  |  |  | |
| Utah | 1.42  (1.03-1.81) | <.001 | 1973-1998 | -0.15  (-0.91-0.62) | .70 | 1998-2014 | 3.38  (2.41-4.37) | <.001 |  |  |  | |
| Washington | 0.95  (0.64-1.25) | <.001 | 1974-1999 | -0.07  (-0.66-0.51) | .80 | 1999-2014 | 2.48  (1.58-3.39) | <.001 |  |  |  | |

a Overall APC was calculated between 1973-2014 for all states except Georgia; 1975-2014, and Washington; 1974-2014

b Annual Percentage Changes, calculated using Joinpoint regression software

c Two-sided P value was calculated using t test to determine the significance of APC change
