# Supplementary material for: Trends in pancreatic adenocarcinoma incidence and mortality in the United States in the last four decades; a SEER-based study
Source: BMC Cancer. 2018 Jun 25;18:688. doi: 10.1186/s12885-018-4610-4 (PMC6020186; doi:10.1186/s12885-018-4610-4)
Supplement: Supplementary file 6 — Trends in Adenocarcinoma of head of pancreas and Adenocarcinoma of body and tail of pancreas Incidence and incidence-based mortality rates (1973-2014). (DOCX 13 kb) [file 12885_2018_4610_MOESM6_ESM.docx]

Additional file 6. Trends in Adenocarcinoma of head of pancreas and Adenocarcinoma of body and tail of pancreas Incidence and incidence-based mortality rates (1973-2014)

|  | Incidence | | | | Incidence-based mortality | | | |
| --- | --- | --- | --- | --- | --- | --- | --- | --- |
|  | Adenocarcinoma of head of pancreas | | Adenocarcinoma of body and tail of pancreas | | Adenocarcinoma of head of pancreas | | Adenocarcinoma of body and tail of pancreas | |
|  | APC^a^  (95% CI) | P value^b^ | APC^a^  (95% CI) | P value^b^ | APC^a^  (95% CI) | P value^b^ | APC^a^  (95% CI) | P value^b^ |
| Overall | 0.87  (0.68-1.07) | <.001 | 3.42  (3.06-3.79) | <.001 | 2.11  (1.73-2.50) | <.001 | 4.31  (3.88-4.74) | <.001 |
| Sex |  |  |  |  |  |  |  |  |
| Male | 0.69  (0.50-0.87) | <.001 | 3.38  (3.01-3.74) | <.001 | 2.21  (1.84-2.58) | <.001 | 4.55  (4.13-4.97) | <.001 |
| Female | 1.02  (0.77-1.27) | <.001 | 3.42  (3.00-3.84) | <.001 | 2.05  (1.63-2.48) | <.001 | 4.07  (3.58-4.57) | <.001 |
| Race |  |  |  |  |  |  |  |  |
| White | 0.99  (0.79-1.19) | <.001 | 3.47  (3.08-3.85) | <.001 | 1.87  (1.48-2.26) | <.001 | 3.98  (3.55-4.41) | <.001 |
| Black | 0.26  (-0.12-0.64) | .17 | 3.04  (2.49-3.59) | <.001 | 2.59  (2.05-3.14) | <.001 | 5.08  (4.45-5.72) | <.001 |
| Others^c^ | 0.44  (0.08-0.80) | .02 | 3.01  (2.30-3.72) | <.001 | 4.49  (3.94-5.05) | <.001 | 6.59  (5.65-7.54) | <.001 |
| Age at diagnosis, y |  |  |  |  |  |  |  |  |
| <60 | 0.32  (0.09-0.54) | .01 | 2.90  (2.42-3.38) | <.001 | 1.15  (1.99-7.64) | <.001 | 3.62  (2.99-4.26) | <.001 |
| >60 | 1.02  (0.81-1.24) | <.001 | 3.60  (3.24-3.95) | <.001 | 2.24  (1.82-2.65) | <.001 | 4.50  (4.07-4.93) | <.001 |
| Stage at diagnosis^d^ |  |  |  |  |  |  |  |  |
| Localized | 0.42  (-0.15-0.99) | .15 | 6.45  (5.32-7.58) | <.001 | 1.08  (0.53-1.63) | <.001 | 3.10  (2.47-3.73) | <.001 |
| Regional | 1.83  (1.58-2.09) | <.001 | 3.03  (2.40-3.67) | <.001 | 2.96  (2.44-3.48) | <.001 | 4.02  (3.22-4.83) | <.001 |
| Distant | 0.63  (0.43-0.83) | <.001 | 3.39  (3.00-3.77) | <.001 | 2.08  (1.76-2.41) | <.001 | 4.61  (4.15-5.06) | <.001 |

a Overall Annual Percentage Changes during 1973-2014, calculated using Joinpoint regression software

b Two-sided P value was calculated using t test to determine the significance of APC change

c Includes American Indian/Alaskan Native and Asian/Pacific Islander.

d using SEER historic stage
