# Supplementary material for: Trends in pancreatic adenocarcinoma incidence and mortality in the United States in the last four decades; a SEER-based study
Source: BMC Cancer. 2018 Jun 25;18:688. doi: 10.1186/s12885-018-4610-4 (PMC6020186; doi:10.1186/s12885-018-4610-4)
Supplement: Supplementary file 7 — Trends in Pancreatic adenocarcinoma Incidence-based mortality Rates by state (1973-2014). (DOCX 13 kb) [file 12885_2018_4610_MOESM7_ESM.docx]

Additional file 7. Trends in Pancreatic adenocarcinoma Incidence-based mortality Rates by state (1973-2014)

|  | Overall  (1973-2014)^a^ | | Trends | | | | | | | | |  |
| --- | --- | --- | --- | --- | --- | --- | --- | --- | --- | --- | --- | --- |
|  |  |  | 1 | | | 2 | | | 3 | | | |
|  | APC^b^  (95% CI) | P value^c^ | year | APC^b^  (95% CI) | P value^c^ | year | APC^b^  (95% CI) | P value^c^ | year | APC^b^  (95% CI) | P value^c^ | |
| Pancreatic adenocarcinoma |  |  |  |  |  |  |  |  |  |  |  | |
| California | 1.31  (1.02-1.60) | <.001 | 1973-2002 | 0.82  (0.45-1.18) | <.001 | 2002-2012 | 3.55  (2.01-5.12) | <.001 | 2012-2014 | -21.04  (-37.29 - -0.59) | .04 | |
| Connecticut | 2.05  (1.69-2.40) | <.001 | 1973-2012 | 2.27  (2.02-2.52) | <.001 | 2012-2014 | -27.77  (-45.87- -3.63) | .03 |  |  |  | |
| Georgia | 3.81  (3.41-4.21) | <.001 | 1975-1994 | 2.75  (1.55-3.96) | <.001 | 1994-2012 | 4.95  (3.86-6.06) | <.001 | 2012-2014 | -15.54  (-38.00-15.06) | .27 | |
| Hawaii | 3.08  (2.67-3.49) | <.001 | 1973-2012 | 3.29  (2.92-3.67) | <.001 | 2012-2014 | -24.73  (-51.59-17.04) | .20 |  |  |  | |
| Iowa | 1.66  (1.35-1.98) | <.001 | 1973-2000 | 1.27  (0.79-1.75) | <.001 | 2000-2012 | 3.13  (1.86-4.43) | <.001 | 2012-2014 | -25.22  (-42.19- -3.26) | .03 | |
| Michigan | 1.67  (1.38-1.96) | <.001 | 1973-1980 | 5.31  (1.30-9.47) | .01 | 1980-2012 | 1.69  (1.44-1.96) | <.001 | 2012-2014 | -18.52  (-33.06- -0.83) | .04 | |
| New Mexico | 2.96  (2.39-2.53) | <.001 | 1973-2003 | 2.36  (1.65-3.09) | <.001 | 2003-2012 | 7.50  (2.72-12.50) | <.001 | 2012-2014 | -30.65  (-49.24- -5.26) | .02 | |
| Utah | 3.81  (3.33-4.30) | <.001 | 1973-2002 | 2.79  (2.17-3.41) | <.001 | 2002-2012 | 7.72  (4.55-10.98) | <.001 | 2012-2014 | -26.59  (-49.60-6.91) | .10 | |
| Washington | 2.86  (2.45-3.28) | <.001 | 1974-2000 | 2.43  (1.76-3.10) | <.001 | 2000-2012 | 4.35  (3.01-5.72) | <.001 | 2012-2014 | -25.62  (-41.59- -5.27) | .02 | |

a Overall APC was calculated between 1973-2014 for all states except Georgia; 1975-2014, and Washington; 1974-2014

b Annual Percentage Changes, calculated using Joinpoint regression software

c Two-sided P value was calculated using t test to determine the significance of APC change
